# A potential therapeutic strategy for prostatic disease by targeting the oral microbiome

**DOI:** 10.1002/med.21778

**Published:** 2020-12-30

**Authors:** Cheng Fang, Lan Wu, Cong Zhu, Wen‐Zhong Xie, Hailiang Hu, Xian‐Tao Zeng

**Affiliations:** ^1^ Center for Evidence‐Based and Translational Medicine Zhongnan Hospital of Wuhan University Wuhan Hubei China; ^2^ Department of Stomatology Zhongnan Hospital of Wuhan University Wuhan Hubei China; ^3^ Department of Urology Zhongnan Hospital of Wuhan University Wuhan Hubei China; ^4^ Department of Stomatology Kaifeng University Health Science Center Kaifeng Henan China; ^5^ Department of Pathology Duke University School of Medicine Durham North Carolina USA; ^6^ School of Medicine Southern University of Science and Technology Shenzhen Guangdong China

**Keywords:** benign prostatic hyperplasia, oral microbiome, periodontal disease, prostate cancer, prostatitis

## Abstract

Nowadays, human microbiome research is rapidly growing and emerging evidence has witnessed the critical role that oral microbiome plays in the process of human health and disease. Oral microbial dysbiosis has been confirmed as a contributory cause for diseases in multiple body systems, ranging from the oral cavity to the gastrointestinal, endocrine, immune, cardiovascular, and even nervous system. As research progressing, oral microbiome‐based diagnosis and therapy are proposed and applied, which may represent potential drug targets in systemic diseases. Recent studies have uncovered the possible association between periodontal disease and prostatic disease, suggesting new prevention and therapeutic treatment for the disease by targeting periodontal pathogens. Thus, we performed this review to first explore the association between the oral microbiome and prostatic disease, according to current knowledge based on published articles, and then mainly focus on the underlying molecular and cellular mechanisms and the potential prevention and treatment derived from these mechanistic studies.

## 1 INTRODUCTION

Benign diseases of the prostate, including prostatitis and benign prostatic hyperplasia (BPH), as well as prostate cancer, account for a large proportion of male urologic disease. Prostatitis has been the most frequent urologic condition in young‐ and middle‐aged men, which is characterized by inflammation of the prostate gland.^1^ The prevalence of prostatitis diagnosed medically is approximated as 9%, and the overall lifetime prevalence in contemporary research is reported to be 14%.^2^ BPH, another common urinary disease, is mainly prevalent in the elder male population, with an increased amount of epithelial and stromal cells in the periurethral area of the prostate.^3^ It is known that the prevalence and diagnostic rate of BPH in males increase with age^4^ and a recent meta‐analysis proposed a prevalence of BPH of 26.2% in a lifetime despite ethnic background.^5^ Prostate cancer, the second most frequently diagnosed cancer among men worldwide, accounting for 7.1% of total cancer cases.^6^ Inflammation has been noticed for years in the process of prostatic diseases and considered to be a possible causal factor in benign disease and prostate cancer.^7^, ^8^ Histological evidence of inflammatory infiltrates has almost been commonly detected in patients with BPH, who nearly all having symptoms of prostatitis.^9^ However, the pathophysiologic mechanisms of the benign prostatic diseases remain incompletely characterized and the relationship between the two disease conditions is still unclear. Numerous epidemiological studies suggest that men with a history of chronic inflammation or prostatitis have increased risks of having prostate cancer.^10^, ^11^ However, the epidemiological links between prostatitis and prostate cancer development remain arguable. Current therapeutic treatments for prostatic disease include pharmacological and surgical therapies. Unfortunately, multidrug‐resistance and post‐surgery adverse effects remain big challenges for these treatments.^12^, ^13^, ^14^ Therefore, determining the key contributors in the involvement of prostate biology is crucial in establishing preventive treatments and therapeutic strategies.

On the basis of findings of the National Institute of Health‐Human Microbiome Project (NIH‐HMP), the association between the human microbiome and health is likely to be a fast‐moving domain in the research area.^15^ To date, the majority of studies^7^, ^16^, ^17^ have tried to investigate the link between genitourinary microbiota and urologic disease, focusing on their roles in pathophysiology and management of these common prostatic dysfunctions. However, indirect internal interactions between the microbiome and prostatic disease, for example, the impacts of the oral or gastrointestinal microbiome on prostatic disease are poorly understood. Herein, we have summarized a constructive table showing the prostatic disease‐associated microbes presented in the prostate gland or prostatic secretion samples from all nine studies^18^, ^19^, ^20^, ^21^, ^22^, ^23^, ^24^, ^25^, ^26^ (Table 1). Except for the microbiota of the genitourinary tract, the oral microbiota deserves more attention according to the strong evidence of epidemiology association between periodontal disease and prostatic disease. Recently, most studies including our findings^27^, ^28^, ^29^, ^30^ have shown that periodontal disease also increases the risk of prostatic disease. The microbial dysbiosis, defined as the change of microbial composition or alteration of bacterial pathogenicity, is important in determining the activity of the periodontal disease.^31^ To control supragingival plaque and prevent disease progression, periodontal treatment adopted a combination of therapeutic interventions and lifelong maintenance of periodontal health, including individually oral hygiene and dietary instructions, removing subgingival plaque and calculus, local and systemic pharmacotherapy, and various types of surgery.^32^ Studies have demonstrated that periodontal treatment can reduce systemic inflammation and markers of endothelial dysfunction within 2–6 months,^33^ which may act as novel therapeutic targets for inflammation‐linked diseases, such as prostatic disease.

**Table 1:** Overview of prostatic disease‐associated microbes presented in the prostate gland or prostatic secretion samples

Author (year)	Country	Study design	Sample size	Sample type	Identification methods	Main outcomes (alterations of the microbiota)	References
Chronic prostatitis (CP)							
Estemalik 2017	USA	Case series	14 CP	Prostatic secretion and dental plaque samples	PCR	(1) A total of 8 of 14 patients with CP had no less than one pathogen in their prostatic secretion samples	^[18]^
						(2) *Porphyromona sgingivali*s, *Treponema denticola*, and *Escherichia coli* were identified in both prostatic secretion samples and dental plaques within the sample individual	
Benign prostatic hyperplasia (BPH)							
Estemalik 2017	USA	Case series	10 BPH	Prostatic secretion and dental plaque samples	PCR	(1) A total of 9 of 10 patients with BPH had no less than one pathogen in their prostatic secretion samples	^[18]^
						(2) *Porphyromonas gingivalis*, *Treponema denticola* and *Escherichia coli* were identified in both prostatic secretion samples and dental plaques within the sample individual	
Jain 2020	India	Case series	36 BPH	Prostate tissues	16S rRNA gene sequencing	(1) Live bacteria were identified in 55.5% (20/36) of the BPH tissues	^[19]^
						(2) *Staphylococcus, Escherichia coli*, and *Micrococcus* spp. were the major isolates	
						(3) *Proteobacteria, Actinobacteria, Firmicutes, and Bacteroidetes were* the most common phylum	
Prostate cancer (PCa)							
Yu 2015	China	Case control	13 PCa and 21 BPH	Prostatic secretions/seminal fluid, voided urine	16S rRNA gene sequencing with PCR‐DGGE analysis	(1) Increased number of Bacteroidetes bacteria, Alphaproteobacteria, Firmicutes bacteria, Lachnospiraceae, *Propionicimonas*, *Sphingomonas*, and *Ochrobactrum* in patients with PCa compared to patients with BPH	^[20]^
						(2) Decreased number of *Eubacterium* and *Defluviicoccus* in patients with PCa compared to patients with BPH	
						(3) *Escherichia coli* in patients with PCa was increased in the prostate secretion and seminal fluid samples and decreased in urine; while *Enterococcus* was increased in the seminal fluid with little change in urine and prostate secretion samples	
Cavarretta 2017	Italy	Case series	16 PCa	Prostate tissues	Ultradeep pyrosequencing	(1) *Propionibacterium* spp. were most abundant at genus level	^[21]^
						(2) *Staphylococcus* spp. were more represented in the tumor/peritumor tissues	
Banerjee 2019	USA	Case control	50 PCa and 15 BPH	Prostate tissues	Array‐based metagenomic and capture sequencing	(1) Diverse microbiome signatures were identified in PCa tissues compared to BPH tissues	^[22]^
						(2) Three prostate cancer‐specific microbiome signatures were identified and related to the stages, grades and scores of PCa	
Feng 2019	China	Case series	22 PCa	Prostate tissues	Metagenomic analysis of host‐derived whole genome sequencing data	(1) *Escherichia*, *Propionibacterium*, and *Pseudomonas were* the most frequent genera	^[23]^
						(2) The core tumor tissues were enriched for *Proteobacteria*	
						(3) African samples were enriched for *Escherichia* and *Acidovorax*, with plentiful *Eubacterium* linked to host tumor hypermutation	
Feng 2019	China	Case series	65 PCa	Prostate tissues	Metagenome and metatranscriptome analyses	(1) *Escherichia, Propionibacterium, Acinetobacter*, and *Pseudomonas* were abundant and the core prostate microbiome	^[24]^
						(2) The microbiome biodiversity could not be differentiated between the tumor and adjacent benign tissues.	
						(3) Ten *Pseudomonas* genes were strongly associated with host small RNA genes; three of which may negatively correlate with cancer metastasis.	
Ma 2019	China	Case control	32 PCa and 27 non‐PCa	Prostatic fluid	16S RNA gene sequencing	(1) The diversity of microbiota was lower in the PCa patients compared to non‐PCa patients	^[25]^
						(2) *Alkaliphilus*, *Enterobacter*, *Lactococcus*, *Cronobacter*, *Carnobacterium*, and *Streptococcus* were different between the two groups	
Miyake 2019	Japan	Case control	45 PCa and 33 BPH	Prostate tissues	PCR	The proportion of *Mycoplasma genitalium* was higher in PCa patients compared to BPH	^[26]^

Abbreviations: PCR‐DGGE, polymerase chain reaction‐denaturing gradient gel electrophoresis; rRNA, ribosomal RNA.

However, there is no review, according to our knowledge, to summarize the association between the oral microbiome and prostatic diseases and the applications of the oral microbiome in the management of prostatic disease. Here, we present this review to summarize the findings related to periodontal disease and prostatic diseases, and then mainly focus on underlying the molecular mechanism roles of the oral microbiome in the diseases, so as to provide potential therapeutic targets. We, therefore, believe this review will be timely and necessary that could both provoke new ideas and promote relevant research and practice on this topic.

## 2 EPIDEMIOLOGICAL AND EXPERIMENTAL EVIDENCE

### 2.1 Oral microbiome and prostatic inflammation

The microflora of the oral cavity is composed of a unique group of bacteria, fungi, viruses, archaea, and protozoa, generally known as the microbiome.^34^, ^35^ The term microbiome encompassing the summary of all microbiotic genetic information, thus, is becoming more popular.^36^ The primary habitats of oral surfaces are predominantly facultative anaerobes such as *Streptococci* and *Actinomyces* species.^37^ Nowadays, more than 1000 different taxonomic bacteria have been proved in the human oral cavity and research employing next‐generation sequencing (NGS) methods demonstrate that the coverage of diversity of oral bacteria maybe even much larger.^38^ Bacteria, the major microorganisms that inhabit the oral cavity, are, therefore, the focus of this review.

Periodontal disease is an inflammatory disorder that refers to the impairment of the supportive tissues around the teeth, which is triggered by the accumulation of bacteria in a microbial biofilm on the teeth and gingiva.^31^, ^39^ Culture‐ and molecular‐based research revealed that the periodontal bacterial pathogens are significantly linked to clinical parameters of periodontal diseases, such as gingival redness, bleeding on probing (BOP), and periodontal destruction. To simplify the description of the microbiota from multiple individual species to complexes of bacteria, the microbial composition of supragingival plaque has been classified into six major complexes: the “red complex” and “orange complex” are predominantly correlated with periodontal disease, the other four complexes (green complex, yellow complex, purple complex, and *Actinomyces* complex) are auxiliary pathogens.^31^, ^40^, ^41^, ^42^ The species of “red complex” comprise *Porphyromonas gingivalis*, *Tannerella forsythia*, and *Treponema denticola*; the “orange complex” was mainly composed of *Fusobacterium nucleatum*, *Prevotella nigrescens*, *Prevotella intermedia*, *Campylobacter rectus*, and *Peptostreptococcus micros*. There is amount of microbiota in the oral cavity, we believe many as‐yet uncultivated phylotypes to be associated with periodontal disease apart from the above six complexes; in fact, emerging oral pathogens are continuous to be identified.^43^, ^44^ Hence, the current “6 major complexes” classification would be modified in the future.

There is compelling evidence that the oral microbiome can extend beyond the oral cavity, and systemic conditions such as Alzheimer's disease, coronary artery disease, arthritis, and colitis are linked to the oral microbiome.^45^, ^46^, ^47^, ^48^ Chronic prostatitis (CP) and BPH are complex inflammatory conditions, however, the relevant etiology of the inflammation is far from being clearly understood. The clinical observation that bacteriuria and urinary tract infections are common sequelae of BPH and the frequent occurrence of CP and BPH together in the aging male may indicate that the chronic inflammation in the prostate is likely caused by chronic transmissible infections.^49^ This inflammatory condition may present a potential novel diagnostic and therapeutic target for these diseases.

Periodontal disease and prostatic diseases are distinct types and seemly unrelated diseases. However, several common risk factors, such as age, metabolic disorders, psychological factors, are all related to diseases.^50^, ^51^, ^52^ Both diseases affect the middle‐aged and elderly people, and long disease course and unsatisfactory treatment results have brought a huge burden to the healthcare system.^5^, ^53^, ^54^ Furthermore, chronic inflammation has been shown to contribute to or associate with the development of both periodontal and prostatic diseases.^7^, ^55^, ^56^ Table 2 summarizes previous epidemiological studies^18^, ^27^, ^28^, ^29^, ^30^, ^57^, ^58^, ^59^, ^60^, ^61^, ^62^, ^63^, ^64^, ^65^, ^66^, ^67^, ^68^, ^69^ exploring the associations between periodontal disease and prostatic disease. Thus, it is of great clinical significance to explore the interaction mechanism between these diseases and whether it affects the development and prognosis of the diseases.

**Table 2:** Epidemiological studies explore the associations between periodontal disease and prostatic disease

Author (year)	Country	Prostate diagnosis	Study design	Sample information	Periodontal examination	Main outcomes	References
Hujoel 2003	USA	PCa	Cohort	11,328 Adults in the NHANES I epidemiologic follow‐up study	Gingival inflammation, periodontal pockets, firmness of tooth	Both periodontitis (OR = 1.81, 95% CI = 0.76–4.34) and gingivitis (OR = 1.48, 95% CI = 0.56–3.94) were associated with increased risk of PCa	^[57]^
Hiraki 2008	Japan	PCa	Case‐control	5240 Cancer subjects and 10,480 controls who visited the Aichi Cancer Center	Teeth number	Tooth loss was inversely associated with the risk of PCa (OR = 0.49, 95% CI = 0.19–1.26)	^[58]^
Michaud 2008	USA	Advanced PCa	Cohort	48,375 US male health professionals in the health professionals follow‐up study	Bone loss, teeth number	(1) No significant association was observed between periodontal disease and advanced PCa risk (HR = 0.89, 95% CI = 0.71–1.10)	^[59]^
						(2) Tooth loss was inversely correlated with advanced PCa (HR = 0.70, 95% CI = 0.50–0.97)	
Arora 2010	Australia (Swedish twins)	PCa	Cohort	15,333 Patients with periodontal disease identified in the Swedish Twin Registry	Tooth mobility	Periodontal disease was correlated with increased risk of PCa (HR = 1.47, 95% CI = 1.04–2.07)	^[60]^
Joshi 2010	USA	CP	Cross‐sectional	35 Subjects underwent prostate biopsy due to abnormal findings on DRE or elevated PSA (≥4 ng/ml)	CAL, GI, PI, GR, BOP, PD, teeth number	(1) PSA levels were obviously greater in patients suffering from moderate/severe prostate inflammation than those none/mild patients	1, 27
						(2) Participants with CAL ≥ 2.7 mm showed higher but not statistically significant levels of PSA	
						(3) Participants with both moderate/severe prostatitis and CAL ≥ 2.7 mm showed significantly greater PSA levels than those with either condition	
Boland 2013	USA	BPH	Case‐control	2475 Patients (1235 cases and 1240 controls) underwent dental treatment	NR	A previously unreported association between periodontitis and BPH (OR = 1.5, 95% CI = 1.05–2.10) after adjusting for confounding factors	^[28]^
Hwang 2014	China	PCa	Cohort	38,902 Patients who received at least 10 treatments for periodontal disease	NR	The treatment cohort had a significantly higher rates of PSA testing and PCa risk (HR = 2.11, 95% CI = 1.63–2.73)	^[61]^
Alwithanani 2015	USA	Prostate inflammation	Cross‐sectional	27 Men underwent prostate biopsy due to abnormal findings on DRE or elevated PSA (≥4 ng/ml)	CAL, GI, PI, GR, BOP, PD	(1) After periodontal treatment, all periodontal parameters and IPSS values displayed statistical improvement	^[62]^
						(2) Men with >4 ng/ml levels of PSA at baseline, displayed obvious reduction in PSA after periodontal treatment.	
						(3) After periodontal treatment, a statistical correlation was observed between the changes in levels of PSA and periodontal parameters (CAL, GI, BOP, and GR)	
Matsumoto 2015	Japan	LUTS	Cross‐sectional	88 Men and 97 women who completed the interview sheet of the CPD and LUTS	NR	(1) Urgency and weak stream score of IPSS were significantly correlated with the severe degree of CPD in both genders	^[63]^
						(2) Significant association between the CPD severity and the OAB presence was only observed in male patients	
Michaud 2016	USA	PCa	Cohort	19,933 Non‐smokers in the health professionals’ follow‐up study	Bone loss, teeth number	(1) There was no obvious relationship between periodontal disease and PCa risk (HR = 1.17, 95% CI = 0.94–1.47)	^[64]^
						(2) No significant association was observed between tooth loss (HR = 0.89, 95% CI = 0.61–1.30) and PCa risk	
Dizdar 2017	Turkey	PCa	Cohort	Patients diagnosed with moderate to severe periodontitis	Clinical and radiographic parameters, that is, CAL, PD	Men with moderate to severe periodontitis had significantly higher risk of prostate cancer (SIR = 3.75, 95% CI = 0.95–10.21)	^[65]^
Estemalik 2017	USA	CP or BPH	Case series	24 Patients diagnosed with CP or BPH (confirmed by DRE) in the urology institute	CAL, PD, BOP, GI, PI, tooth mobility	(1) A total of 8 of 14 patients with CP and 9 of 10 patients with BPH and had no less than one oral pathogen in their prostatic secretion samples	^[18]^
						(2) *Porphyromonas gingivalis*, *Treponema denticola*, and *Escherichia coli* were found in both prostatic secretion samples and dental plaques	
Kruck 2017	Germany	Asymptomatic	Prospective trial	47 Asymptomatic men presenting for periodontal treatment	BOP, dental status, furcation involvement, vertical bone loss, pocket secretion	(1) None of the periodontal parameters measured at baseline showed an association with tPSA levels	^[66]^
						(2) PSA testing before and after CP treatment revealed no significant differences in tPSA, fPSA, or %PSA	
Lee 2017	South Korea	PCa	Cohort	A stratified sample of 187,934 participants collected from the NHIS database	CAL, PD, BOP, gingival inflammation and alveolar bone loss	Patients with periodontal disease has 14% higher risk of PCa (HR = 1.14, 95% CI = 1.01–1.31)	^[67]^
Boyapati 2018	India	CP	Cross‐sectional	100 Patients with CP diagnosed to also have periodontal diseases	CAL, PD, BI, GI, PI	(1) The mean CAL was obviously higher in patients suffering moderate‐to‐severe prostatitis than mild prostatitis patients	^[68]^
						(2) A significantly positive association was found between PSA and periodontal parameters (CAL scores, PD scores, GI and PI values)	
Güven 2019	Turkey	PCa	Cohort	5199 Patients with periodontal disease	Clinical and radiographic parameters	Periodontal diseases significantly increased the risk of PCa (SIR = 1.84, 95% CI = 1.34–2.49) in men	^[30]^
Huang 2019	USA	Asymptomatic	Cross‐sectional	1263 Men participated in the National Health and Nutrition Examination Survey in 2009–2010	GR, PD, CAL, teeth number	(1) The PSA levels did not change with severity of periodontal disease compared to those without disease after adjusting for confounding factors	^[69]^
						(2) Participants with higher severity of periodontal disease were more able to posses PSA > 4.0 ng/ml compared with those free from periodontal disease, although the adjusted OR was insignificant	
Wu 2019	China	BPH	Cross‐sectional	2171 Male participants were selected from a health examination	CPI	(1) Periodontal disease obviously raised the risk of BPH (OR = 1.68, 95% CI = 1.26–2.24), and subjects with periodontitis displayed a higher risk (OR = 4.18, 95% CI = 2.75–6.35) after adjusting for confounding factors	^[29]^
						(2) Stratified analysis showed that periodontal disease significantly increased higher risk in the group with a prostate volume > 60 g	

Abbreviations: BI, bleeding index; BOP, bleeding on probing; BPH, benign prostatic hyperplasia; CAL, clinical attachment level; CI, confidence interval; CP, chronic prostatitis; CPD, chronic periodontal disease; CPI, community periodontal index; DRE, digital rectal examination; fPSA, free PSA; GI, gingival index; GR, gingival recession; HR, hazard ratio; IPSS, international prostate symptom score; LUTS, lower urinary tract symptoms; NHANES, National Health and Examination Study; NHIS, National Health Insurance Service; NR, not reported; OAB, overactive bladder syndrome; OR, odds ratio; PCa, prostate cancer; PD, probing depth; PI, plaque index; PSA prostate‐specific antigen; SIR, standardized incidence rate; tPSA, total PSA; %PSA, fPSA/tPSA ratio.

### 2.2 Periodontal disease, *porphyromonas gingivalis*, and prostatitis

In terms of the NIH classification, prostatitis is grouped into four categories as follows: category I, acute bacterial prostatitis; Category II, chronic bacterial prostatitis; Category III, CP/chronic pelvic pain syndrome; and Category IV, asymptomatic inflammatory prostatitis.^70^ Men suffered from CP always presenting the symptoms of perineal or pelvic pain, and some patients also experience dysfunctional voiding symptoms.^71^, ^72^ The prostate is susceptible to infections by pathogenic microorganisms in the male reproductive system. Gram‐negative bacteria, such as *Escherichia coli* and *Klebsiella spp*, were involved in the etiology of prostate inflammation.^27^, ^73^ As noted above, Gram‐negative bacteria (i.e., *Porphyromonas gingivalis)* also act as etiologic agents for periodontal disease.

A recent study^68^ involving 100 patients diagnosed with CP and periodontal diseases found that the mean clinical attachment level (CAL) was obviously higher in males suffered from moderate‐to‐severe prostatitis than mild prostatitis patients, which provides a possible pathological correlation between the two diseases. Furthermore, Estemalik et al.^18^ identified oral pathogens from the patients’ prostatic secretions and dental plaque, who suffered periodontal disease and CP or BPH simultaneously using polymerase chain reaction (PCR). They found that 17 (70.8%) patients have gained no less than one oral pathogen in their prostatic secretions. A total of 8 of 14 (57.1%) prostatic secretion samples of patients with CP contained at least one studied oral pathogens. *Porphyromonas gingivalis* was isolated from both dental plaques and prostatic secretions in 6 of 17 (35.3%) patients, and *Treponema denticola* was detected in 7 of 15 (46.7%) patients. Taken together, it is indicated that periodontal pathogens might play a certain role in correlating the development of periodontal disease and benign prostatic disease.

### 2.3 Periodontal disease, *porphyromonas gingivalis*, and BPH

BPH, the most frequent urologic condition afflicting elder males, is a popular noncommunicable disease (chronic noncommunicable diseases [CNCDs]) in humans.^3^, ^7^, ^74^ It is substantially a histological diagnosis, which is usually clinically defined on the basis of symptoms (lower urinary tract symptoms, LUTS), due to mechanical pressure on the bladder and urethra.^75^

A previous study conducted in the USA demonstrated that individuals suffering from periodontal disease could enhance the risk of BPH by 1.50 times.^28^ Our recent study^29^ on 2171 participants also showed an increased risk of BPH (odds ratio = 1.68, 95% confidence interval = 1.26–2.24) in men with periodontal disease, and patients suffering from periodontitis displayed a higher risk. A significant association was also observed when adjusted by age, body mass index, or blood pressure status, which would strongly suggest a relationship between the two diseases. Furthermore, periodontal disease obviously increased greater risk in the patients whose prostate volume greater than 60 g. Moreover, Matsumoto et al.^63^ investigated the association between chronic periodontal disease and LUTS, and confirmed that urgency and weak stream score of international prostate symptom score (IPSS) was obviously associated with the severity of chronic periodontal disease in both genders. Also, a significant association between the severity of periodontal disease and the existence of overactive bladder syndrome was observed in male patients. Estemalik et al.^18^ also investigated the oral pathogen in patients with BPH, and found that 9 of 10 patients with BPH presented no less than one oral pathogen in their prostatic secretion samples, such as *Porphyromonas gingivalis* and *Treponema denticola*. There was a bit of pity that the study only focused on the detection of three well‐recognized periodontal bacteria. In addition, a detailed and comprehensive analysis of the microbial diversity (e.g., abundance) has not been reported in this study, because the authors only used PCR. Therefore, we suggest performing a study using both sequencing analysis and PCR to obtain proportion and microbial diversity, which can confirm the link between the two diseases better.

### 2.4 Periodontal disease and prostate‐specific antigen

Prostate‐specific antigen (PSA), synthesized and secreted by the epithelial cells of the prostate acini, is characterized as an inflammatory marker.^76^ It is most frequently employed for prostate cancer screening despite other interventions or exposure can also lead to increased levels of PSA, such as digital rectal examination, prostate biopsy, prostatitis, and BPH.^77^, ^78^ Numerous studies^79^, ^80^ have revealed that prostatic inflammation contributes greatly to elevated serum levels of PSA.

Previously, Joshi et al.^27^ had studied the correlation between PSA and periodontal disease in CP populations and found that patients with CAL ≥ 2.7 mm and concurrent moderate/severe prostatitis displayed enhanced PSA levels than those with one of these conditions. Consistent with the above results, the recent study^68^ involving subjects with CP and periodontal diseases observed a significant and positive correlation between PSA and periodontal parameters, including CAL scores, probe depth (PD) scores, gingival index (GI), and plaque index values. Whereas, in a cross‐sectional study of 1236 men without prostate cancer diagnosis or inflammation or infection of the prostate, the PSA levels did not increase in periodontal disease patients when taken age and other factors into account. Interestingly, patients with higher severity of periodontal disease were more probably to possess PSA greater than 4.0 ng/ml compared with those free from periodontal disease, although the multivariable‐adjusted ORs revealed insignificant.^69^ Moreover, Alwithanani et al.^62^ reported that periodontal treatment reduced the PSA value in men afflicted with abnormal prostate on digital rectal examination. A statistical correlation between the alteration of PSA levels and periodontal parameters, such as CAL, GI, BOP, and gingival recession, was also observed after periodontal treatment. However, in a prospective trial of 47 chronic periodontitis patients who did not receive any prostate diagnostics or treatment, the periodontal treatment showed no impacts on PSA reduction.^66^ Taken together, the findings above suggest that PSA levels may be correlated with the severity degree of periodontal disease in participants with symptomatic prostatic disease. Whereas, such a potential link seems to be excludable in asymptomatic men. Of course, prospective research with a large sample size should be designed to further explore the relationship.

### 2.5 Possible mechanisms

Increasing evidence supports that bacterial infections, inflammatory stimuli, and environmental factors are considered key contributors to the development of prostatic diseases.^21^, ^81^ Basic experimental studies have shown that oral bacterial infections can stimulate the inflammatory response, thus affecting cell apoptosis and accelerating certain systemic diseases.^82^, ^83^ Although the relationship between oral microbiome with prostate health has been linked, the underlying mechanisms remain most elusive.

### 2.6 Direct pathway

#### 2.6.1 Distant dissemination of oral pathobionts

Links between the oral microbiome and systemic disease have gained increasing attention. For instance, several periodontal pathogens were detected in atherosclerotic plaques, including *Porphyromonas gingivalis*, *Aggregatibacter actinomycetemcomitans*, and *Tannerella forsythia*.^46^ Similarly, Témoin et al.^47^ detected the oral bacteria DNA from synovial fluid of subjects with arthritis. The authors suggested that oral pathogens had moved to the extraoral site through the bloodstream. As mentioned above, Estemalik et al.^18^ isolated *Porphyromonas gingivalis* and *Treponema denticola* from patients’ dental plaque and prostatic fluid simultaneously. Thus, we purposed that the oral pathogens presented within the same individual may spread from their regular residence and arrived at the prostate gland via hematogenous spread. These microbes might play the same roles as in the oral cavity once they reach the prostate, resulting in a histological alteration in the organ.

### 2.7 Indirect pathway

#### 2.7.1 Inflammation

There is substantial evidence to establish that periodontal disease leads to amplified levels of proinflammatory cytokines such as interleukin (IL)‐1β, IL‐6, IL‐8, tumor necrosis factor‐α, and interferon‐γ.^56^, ^68^ These cytokines keep the whole body in a chronic weak inflammatory state for a long time and stimulate the occurrence of systemic diseases.

The effect of these proinflammatory cytokines in the ontogenesis of the prostatic disease has also been recognized, therefore making it possible for periodontal disease to indirectly relate to prostate inflammation through the diffusion of cytokines.

As is reported, *Porphyromonas gingivalis* and other periodontitis‐associated bacteria were highly presented in atherosclerotic plaques and might enhance the persistence of inflammation.^84^ Thus, it is highly likely that oral pathogens may initiate or sustain a prostatic inflammatory response, which eventually results in the progression of prostatic disease. The presence of similar oral pathogens, as noted above within prostatic secretions in males with both periodontitis and prostatitis or BPH, further emphasizes the association of both dysfunctions and the possible effect of a common inflammation process. Several animal models^85^, ^86^, ^87^, ^88^, ^89^ have been developed to clarify the effects of chronic bacterial (or abacterial) prostate inflammation on prostate cancer development. The findings reveal that chronic inflammation induced by a bacterial stimulus can trigger an inflammatory response that may persist for several months.^88^, ^89^ A 25‐week follow‐up study by Elkahwaji et al.^90^ using an animal model of CP induced by *Escherichia coli* infection, found it was related to the development of prostate hyperplasia and dysplasia. Moreover, bacterial infection‐induced prostate inflammation could promote basal‐to‐luminal transition in the mouse prostate, probably generating prostate cancer precursor lesions, such as prostatic intraepithelial neoplasia.^89^ Further studies are warranted to better characterize the role of periodontal pathogens in the initiation of carcinogenesis in the prostate gland.

#### 2.7.2 Metabolism

**Figure 1:**
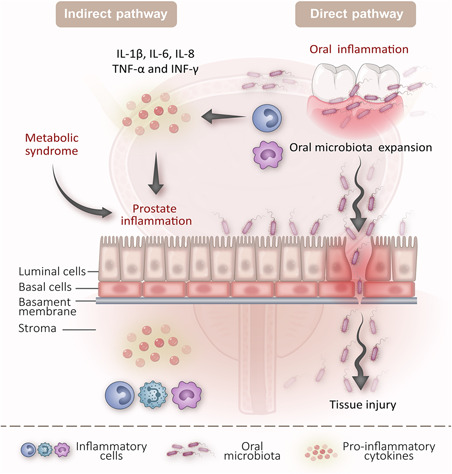
The potential mechanism role of oral microbiota in prostatic disease. The oral microbiota may disseminate from the oral cavity and reached the prostate gland via the hematogenous spread, causing direct tissue injury. Meanwhile, oral pathogens may indirectly induce the prostatic inflammatory process through the dissemination of proinflammatory cytokines, such as IL‐1β, IL‐6, IL‐8, TNF‐α, and IFN‐γ. Then, prostate inflammation could be auto‐sustained or exacerbated by a metabolic disorder, thus contributing to prostatic disease. IFN‐γ, interferon‐γ; IL, interleukin; TNF‐α, tumor necrosis factor‐α [Color figure can be viewed at wileyonlinelibrary.com]

Epidemiology study has revealed the link between periodontal disease and metabolic disorders.^91^ A recent study has demonstrated that patients suffering from metabolic syndrome are at an increased risk of BPH.^92^ Moreover, metabolic syndrome is correlated with larger prostate size, thus supporting the role of metabolic derangements in the pathogenesis and development of a benign prostatic enlargement.^93^ Available evidence further showed that bacterial infection, which could be autosustained or exacerbated by a metabolic disorder, might induce prostatic inflammation. The mechanical obstruction, accompanied by the abnormal metabolic and hormonal milieu on the neck of the bladder, contributing to BPH/LUTS.^51^ It is interesting to note that chronic inflammation might exhibit a common relationship between metabolic syndrome and BPH development.^51^ Metabolites, such as short‐chain fatty acids are commonly produced by gut microbiota during fermentation of dietary fiber, which have similar chemical structures as hormones.^94^ Since hormones are essential for the growth and survival of prostate cells, metabolites may be correlated with prostate growth. Moreover, SFCAs were reported to appear to enhance regulatory T‐cell frequency in the gut, which may influence immune surveillance and involved in carcinogenesis.^95^ Thus, we proposed that the oral pathogens may translocate to the prostate via hematogenous spread and influence the proliferation and apoptosis of the prostate based on inflammatory and metabolism mechanisms (Figure 1).

#### 2.7.3 Immune dysregulation

If the microenvironment of the oral cavity is dysbiotic, immune dysfunction may be induced and the person could suffer periodontal disease, the oral microbiome plays an important role in this immune response.^96^ Of these microorganisms, the *Porphyromonas gingivalis* was reported to trigger oral inflammation and lead to immune dysregulation in mice^97^; alterations of the immune response in the mediation of periodontal disease and cancer were also revealed in other published studies.^98^, ^99^, ^100^ Accordingly, oral dysbiosis may be overall immunosuppression instead of causing prostatic diseases. The evidence between oral microbiome and efficacy of cancer therapy lacked, but the gut microbiome was found to impact the therapeutic efficacy of certain cancer immunotherapy, including cyclophosphamide,^101^ anti‐PD‐L1,^102^ and CTLA‐4 blockade.^103^ It is speculated that the microbiome may serve as a therapy target to be modulated to enhance treatment responses. Due to the lack of relevant studies, we suggest carrying out human or animal research to investigate the interplay between the oral microbiota and immunotherapy response in prostate cancer, also its mechanisms.

## 3 PREVENTION AND TREATMENT

### 3.1 Periodontal intervention and changes in prostatic symptoms

As mentioned above, the study by Alwithanani et al.^62^ reported a periodontal treatment reduced the PSA levels in men afflicted with abnormal prostate on digital rectal examination. The improvement of IPSS after periodontal treatment was also noticed in this study. The authors suggest that rebuilding the integrity of the glandular epithelium of the prostate may decrease the leakage of PSA to enter the blood. Such linkage warrants further investigation since periodontal treatment may eliminate certain periodontal pathogens.

### 3.2 Manipulation of the oral microbiota in early adult

In light of the findings described above, the manipulation of the oral microbiota composition may be proposed as a novel approach to reduce prostatic inflammatory conditions and prevent prostatic disease. A recent systematic review^104^ has summarized the clinical effect of probiotic when it was performed to nonsurgical periodontal treatment as adjunctive therapy and demonstrated that adjunctive use of *Lactobacillus reuteri* showed significant impacts on the improvement of periodontal parameters and reduction of gingival inflammation. Furthermore, probiotic (i.e., *Akkermansia muciniphila*) treatment has demonstrated an inhibitory effect towards *Porphyromonas gingivalis*‐induced periodontal destruction and inflammation.^105^, ^106^ Considering these facts, manipulation of the oral microbiota in an adult may effectively prevent the onset of periodontal and systemic disease. Thus, it is of particular importance to investigate the prevalence of oral microbes in both periodontal and prostatic patients, which may offer an important approach for the prevention and treatment of periodontal and prostatic disease.

### 3.3 Periodontal and prostatic synchronous treatment

As mentioned above, the periodontal treatment improved prostate symptom score and lowered PSA value in men afflicted with chronic periodontitis and prostatic disease. Periodontal disease can influence systematic disease and vice versa. It is known that certain microbiome has the ability to catabolize estrogens and androgens, thereby affecting hormones levels.^107^, ^108^ Although the causal relationship between periodontal disease and prostatic disease remains unclear, synchronous treatment for the patients with periodontal and prostatic diseases may be proposed as a novel strategy in certain situations and it is vital to further explore and understand the relationships that are involved.

### 3.4 Prognosis and self‐management of patients

The main objectives of prostatic disease treatment are to alleviate symptoms, improve life quality, and promote the recovery of related functions. As for BPH, simultaneous therapy using alpha‐blockers and 5‐alpha‐reductase inhibitors has obviously decreased disease progression, whereas, 12.6% of patients show clinical progression after 4‐year treatment, and 5% needing surgical intervention.^13^ It has been indicated that periodontal disease can be successfully treated by active periodontal therapy (APT) and regular obedience during periodontal maintenance therapy (PMT) is important to maintain the remission of periodontal condition gained after APT.^109^, ^110^ In a 6‐year follow‐up prospective study^111^ involving 212 participants in PMT, the author reported that patient compliance positively influenced the composition of subgingival microbes and the steady‐state of periodontal condition. Since oral microbiota may act as potential targets not only for periodontal disease but also for prostatic disease. Thus, receiving PMT and improving patients’ awareness of oral healthcare may have a positive impact on the prognosis of the two diseases.

## 4 PERSPECTIVE AND FUTURE DIRECTIONS

**Figure 2:**
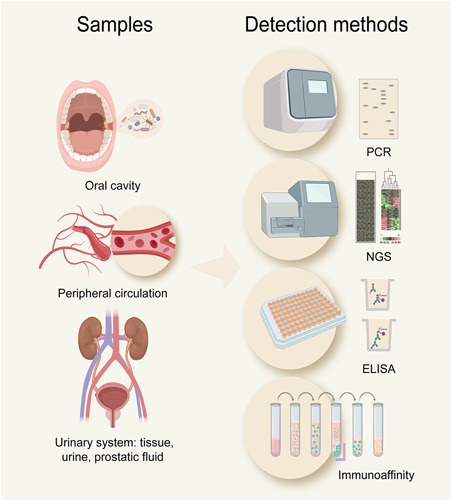
Detection methods for oral microbiota in patients with prostatic disease. The oral microbiota in the oral cavity, peripheral circulation, and urinary system can be detected and quantified using polymerase chain reaction (PCR) and next‐generation sequencing (NGS) detection methods. Multifunctional nanomaterials (i.e., immunomagnetic beads) can be efficiently used to separate oral microbiota and enhance the accuracy of PCR or NGS detection. The specific antigens or antibodies of oral microbiota in blood samples can be detected by enzyme‐linked immunosorbent assay (ELISA) [Color figure can be viewed at wileyonlinelibrary.com]

Current epidemiological studies have demonstrated that periodontal disease is related to prostatic disease. The oral microbiota in the oral cavity, peripheral circulation, and urinary system (including tissue, urine, and prostatic fluid) can be detected and quantified using PCR and NGS detection methods. Furthermore, multifunctional nanomaterials, like immunomagnetic beads, can be efficiently used to separate oral microbiota from peripheral circulation or urinary system and enhance the accuracy of PCR or NGS detection. Better early‐stage diagnosis or prognosis models based on oral microbiota may be established for prostatic disease, providing new alternative opportunities for disease management. In addition to utilizing the information in PCR or NGS data from the oral microbiome, enzyme‐linked immunosorbent assay can also be used to detect the specific antigens or antibodies of oral microbiota in blood samples (Figure 2).

## 5 CONCLUSION

Although correlations between oral microbial dysbiosis and various diseases have been reported in both experimental animals and humans, the mechanistic role of oral microbiota in disease progression remains to be elucidated. In recent years, the epidemiology correlation between periodontal disease and prostatic disease has attracted increasing attention despite controversies still exist in these studies due to limited sample size, different diagnostic criteria for diseases, and various ethnic backgrounds of included populations. The causative relationship between the oral microbiome and prostatic disease, although needs to be solidly established at the molecular level, would have important consequences for disease prevention and therapy. Prospective trials to estimate the preventive impacts of periodontal therapy on prostatic disease should be designed. Ideally, we try to find the specific oral bacteria, but current evidence support that multiple species of microorganism can induce prostatic inflammation, including the known pathogens (i.e., *Escherichia coli*) that causing symptomatic bacterial prostatitis (Table 1), none of them is unique. Furthermore, animal studies report that prostatic inflammation persists for months or years even after clearance of infectious pathogens. Thus, it is difficult to identify a unique infectious microorganism as a causative agent of chronic inflammation in the prostates or as a driver of prostate carcinogenesis. In fact, the microbes that are linked with inflammation in the prostate gland could be further connected with BPH and cancer; the definite mechanisms that remain need to be explored. We also suggest performing studies to investigate which periodontal pathogens involved in and the underlying mechanisms of carcinogenesis in the prostate gland. Furthermore, modulation of oral microbiota has emerged as a potential intervention that could provide a novel strategy to manage periodontal diseases and their systemic effects. With further investigation into the impacts of the oral microbiome on prostatic disease, novel therapies for these diseases will be achieved.

## CONFLICT OF INTERESTS

The authors declare that there are no conflict of interests.
